# Betulinic acid synergically enhances BMP2-induced bone formation via stimulating Smad 1/5/8 and p38 pathways

**DOI:** 10.1186/s12929-016-0260-5

**Published:** 2016-05-17

**Authors:** Hyuck Choi, Byung-Chul Jeong, Min-Suk Kook, Jeong-Tae Koh

**Affiliations:** Research Center for Biomineralization Disorders, School of Dentistry, Chonnam National University, Gwangju, Republic of Korea; Animal Nutrition Physiology Team, National Institute of Animal Science, Rural Development Administration, Wanju-gun, Republic of Korea; Department of Pharmacology and Dental Therapeutics, School of Dentistry, Chonnam National University, Gwangju, Republic of Korea; Department of Oral and Maxillofacial Surgery, School of Dentistry, Chonnam National University, Gwangju, South Korea

**Keywords:** Betulinic acid, BMP2, Bone formation, Mineralization, Smad1/5/8, p38

## Abstract

**Background:**

Healing of bone defects is a dynamic and orchestrated process that relies on multiple growth factors and cell types. Bone morphogenetic protein 2 (BMP2) is a key growth factor for bone healing, which stimulates mesenchymal stem cells to differentiate into osteoblasts. Betulinic acid (BetA) is a natural pentacyclic triterpenoid from plants. This study aimed to examine combinatory effects of BetA and BMP2 on ectopic bone generation in mice.

**Results:**

In MC3T3-E1 preosteoblast culture, 10–15 μM of BetA increased the alkaline phosphatase (ALP) activity and expression levels of osteogenic marker genes without the decreased cell viability. In addition, BetA synergistically enhanced BMP2-induced gene expressions and mineralization with the enhancement of phosphorylation of Smad1/5/8 and p38. In an in vivo ectopic bone formation model, combination of BetA (50 μg) and BMP2 (3 μg) resulted in increases in the amount of new bone generation, compared with treatment with BMP2 alone. Histological studies showed that bone generation with cortical and trabecular structures was resulted from the combination of BetA and BMP2.

**Conclusion:**

BetA can enhance in vivo osteogenic potentials of BMP2, possibly via stimulating Smad 1/5/8 and p38 pathways, and combination of both agents can be considered as a therapeutic strategy for bone diseases.

**Electronic supplementary material:**

The online version of this article (doi:10.1186/s12929-016-0260-5) contains supplementary material, which is available to authorized users.

## Background

An insufficient amount of bone is still considered to be one of the major problems associated with orthopedic procedures or neurosurgery as well as oral and maxillofacial surgery, and various approaches are being developed to overcome this problem [1, 2]. At present, autologous bone graft surgery is a frequently employed approach, which is used to supplement the missing bone. However, autografts require additional invasive procedures and are often painful with limited access to the graft site. Additional complications may include infection, delayed time to healing, morbidity to the donor site as well as the high cost of an operation [3]. In order to address these drawbacks, several families of drugs designed to up-regulate bone formation and increase bone mass have been developed [4]. In a few of Asian countries such as Korea, China and Japan, herbal medicines have been widely investigated for their clinical potential to treat bone disease [5, 6]. In fact, some herbal extracts have demonstrated their bone-forming or osteoprotective effects in cell cultures or animal models [5–7].

Betulinic acid (3β, hydroxy-lup-20 (29)-en-28-oic acid; BetA) is a naturally occurring pentacyclic triterpenoid found in many kinds of fruits, vegetables and most abundant in the *Sambucus williamsii* Hance tree [8, 9]. In China, stem and ramulus of the tree has been used traditionally as an herbal remedy for osteoporosis, joint diseases and bone fracture [10]. BetA has been introduced to have anti-inflammatory and anti-cancer activities in various experimental models [11, 12]. A recent study revealed that BetA also has potent action on osteoblast differentiation and mineralization by the BMP/Smad/Runx2 signaling pathways [9]. However, the study only demonstrated that BetA positively influenced osteogenic functions in MC3T3-E1 preosteoblasts in an in vitro model.

Some growth factors including vascular endothelial growth factor (VEGF), transforming growth factor beta (TGF-β), platelet-derived growth factor (PDGF), and bone morphogenetic proteins (BMPs) are highly expressed during the bone healing cascade [13, 14]. The bone morphogenetic protein 2 (BMP2) has been well-established as a very strong inducer of osteogenesis that plays a critical role during bone formation and remodeling [15]. Furthermore, animal and human studies have shown that treatment with BMP2 results in efficacious bone regeneration and healing with restoration of function [16–18]. However, although the use of BMP2 enhances bone fracture healing, the physiological disadvantages of a short biological half-life combined with rapid local clearance and the requirement for high doses might lead to adverse effects such as osteoclastogenesis, immunological reactions, and edema [14, 19–22]. In addition, the BMP2 protein is not only costly but also undergoes rapid degradation after an initial burst release. In order to minimize risks and reduce the dose required, the effect of BMP2 on bone formation has been investigated in conjunction with a variety of factors including chemical agents, stem cells and genes [13, 23, 24]. Moreover, a combination therapy of anti-osteoporotic agents with BMPs has also been evaluated in the repair of bone defects and induced bone formation [13].

This study investigated the effect of a combination of BetA and BMP2, as compared to BMP2 alone, on ectopic bone formation in mice. We hypothesized that combined delivery of BetA and BMP2 could enhance bone formation and better stimulate the bone healing effects. Our data showed a potential synergy in vivo with a combination of BetA and BMP2; the combination significantly enhanced ectopic bone formation compared to an implant with both single factor, and enhanced osteogenic differentiation through activation of phosphorylated Smad1/5/8 and p38 signaling pathways.

## Methods

### Recombinant proteins and materials

Betulinic acid (BetA) was purchased from Sigma-Aldrich (St. Louis, MO, USA) and dissolved in 0.1 % DMSO. Recombinant human bone morphogenetic protein 2 (BMP2) was purchased from Cowellmedi (Seoul, Korea). The absorbable collagen sponge CollaDerm was obtained from Bioland (Ochang, Korea).

### Cell cultures

MC3T3-E1 pre-osteoblasts were cultured in α-minimal essential medium (α-MEM; Invitrogen, Carlsbad, CA, USA) supplemented with 10 % fetal bovine serum (FBS; Gibco-BRL, Grand Island, USA) and 1 % penicillin/streptomycin (Invitrogen), and incubated at 37 °C in a humidified atmosphere of 5 % CO_2_. For osteogenic differentiation, 50 μg/ml ascorbic acid and 5 mM β-glycerophosphate were added into the culture medium in the presence or absence of BetA and/or BMP2.

### Cell viability assay

The WST-1 assay kit (EZ-CytoX, Daeil Lab Service CO., Seoul, Korea) was used according to the manufacturer's instructions. Briefly, cells were seeded into 96-well plates and incubated in serum-free medium for 24 h. Cells were then stimulated for 24 h with BetA at concentrations ranging from 5 μM to 30 μM, and assays were performed by adding WST-1 directly to the culture. The absorbance of the wells was measured at 540 nm by using spectrophotometry (Multiskan GO, Thermo Scientific, Waltham, USA).

### Total RNA extraction and RT-PCR

Total RNA was isolated from the cultured cells using TRIzol reagent (Invitrogen) according to the manufacturer’s instructions and cDNA was synthesized from the extracted total RNA using random primers and reverse transcriptase (Invitrogen). PCR was then used to amplify the osteogenic markers; mouse alkaline phosphatase (ALP), mouse bone sialoprotein (BSP), mouse osteocalcin (OC), mouse bone morphogenetic protein 2 (BMP2), and mouse glyceraldehyde-3-phophate dehydrogenase (GAPDH). The primer sequences used for PCR amplification were as follows: ALP (F) 5′-TACATTCCCCATGTGATGGC-3′ and (R) 5′-ACCTCTCCCTTGAGTGTGGG-3′, BSP (F) 5′-GGAGGGGGCTTCACTGAT-3′ and (R) 5′-AACAATCCGTGCCACCA-3′, BMP2 (F) 5′-AGCTGCAAGAGACACCCTTT-3′ and (R) 5′- CATGCCTTAGGGATTTTGGA-3′, OC (F) 5′-CTCCTGAGTCTGACAAAGCCTT-3′ and (R) 5′-GCTGTGACATCCATTACTTGC-3′, and GAPDH, (F) 5′-ACCACAGTCCATGCCATCAC-3′ and (R) 5′-TCCACCACCCTGTTGCTGTA-3′. Each reaction consisted of an initial denaturation step at 94 °C for 1 min, followed by a three-stage cycle: denaturation at 94 °C for 30 s, annealing at a temperature optimized for each primer pair for 30 s, and extension at 72 °C for 30 s. After the requisite number of cycles (25–30 cycles), the reactions underwent a final extension at 72 °C for 5 min.

### Alkaline phosphatase (ALP) activity and matrix mineralization

For ALP activity, cultured cells were fixed with 70 % ethanol, rinsed three times with deionized water, and then treated for 15 min with BCIP^®^/NBT solution (Sigma Aldrich, St. Louis, MO, USA). The stained cultures were photographed. For matrix mineralization analysis, alizarin red staining was performed using the manufacturer’s protocols. Briefly, cultured cells were fixed with 70 % ethanol for 60 min, rinsed three times with deionized water, and reacted with 40 mM alizarin red stain solution (pH 4.2) for 15 min. After washing with phosphate-buffered saline (PBS) for 15 min, the stained cultures were then photographed. To quantify the matrix mineralization, stains were extracted with 10 % (w/v) cetylpyridinium chloride and the absorbance was measured against a standard solution by using a spectrophotometer (Thermo Scientific) at 540 nm.

### Western blot analysis

The MC3T3-E1 preosteoblast cells were cultured in medium supplemented with BetA (10 μM) and/or BMP2 (200 ng/ml). After 5 to 10 min, cells were harvested in a lysis buffer (Cell Signaling, Beverly, MA, USA) and the protein concentration was determined by the BCA assay reagent (Bio-Rad Laboratories, Hercules, CA, USA). Proteins were resolved on 10 % SDS-PAGE and transferred to a PVDF membrane. After blocking in 5 % skim milk in Tris-buffered saline with 0.1 % Tween-20 (TBS-T), the membrane was incubated overnight at 4 °C with specific primary antibodies for phospho-Smad1/5/8, total Smad, phospho-Erk1/2, total Erk1/2, phospho-p38, total p38 (Cell Signaling, Beverly, MA, USA) diluted 1:1,000 in 5 % skim milk in TBS-T. After washing, the blots were incubated for 2 h with anti-rabbit horseradish-peroxidase-conjugated antibody (Promega, Madison, WI, USA) diluted 1:3,000 in TBS-T. Signals were detected by an enhanced chemiluminescence reagent (Santa Cruz Biotech, CA, USA) and a LAS-4000 lumino-image analyzer system (Fujifilm, Tokyo, Japan).

### Animal surgery and experimental design

All animal studies were reviewed and approved by the Animal Ethics Committee of Chonnam National University (No. CNU-IACUC-YB-2014-35). Six week-old male C57BL/6 mice (Daehan Biolink, Eumseong, Korea; 20 to 25 g body weight) were divided randomly into five groups (five mice per group). After inducing general anesthesia by intraperitoneal injection of a mixture of Zoletil (30 mg/kg; Virbac Lab, Carros, France) and Rompun (10 mg/kg; Bayer Korea Ltd, Ansan, Korea) each mouse was shaved, and the dorsum prepped with povidone-iodine (Sung Kwang Pharm Ltd, Cheonan, Korea) and ethanol. A sagittal incision (0.8 to 1.0 cm) was made on the back, and then a subcutaneous pocket was formed using blunt dissecting.

Absorbable stable hemostatic collagen sponges (Colladerm, Bioland, Ochang, Korea) cut into pieces (approximately 10 × 10 × 3 mm^3^) under semi-sterile conditions, were used as a carrier to deliver BetA and BMP2. For the control group, the sponges were impregnated with 50 μl of 0.1 % DMSO; for the BMP2 group, 3 μg of BMP2; for the BetA group, 25 or 50 μg of BetA; for the first combination group, 3 μg of BMP2 and 25 μg of BetA; and for the second combination group, 3 μg of BMP2 and 50 μg of BetA. All additions were brought to a total volume of 100 μl in PBS and the sponges were implanted into the subcutaneous pocket.

The animals were sacrificed by CO_2_ asphyxiation at 4 weeks after the implantation. Specimens were carefully dissected and harvested from each group, fixed in 10 % neutral-buffered formalin solution for 24 h, and subsequently transferred into 70 % ethyl alcohol for further radiographic and histology studies.

### Microradiographic analyses

Prior to scarifying the mice, ectopic bone formation in the body was monitored by using a two-dimensional Soft X-ray apparatus (Hitex Ltd, Osaka, Japan) and a diagnostic X-ray film (X-OMAT V, Kodak, Rochester, NY, USA) under the following conditions; 35 kVp and 400 μA for 45 s. For three-dimensional analysis, each isolated specimen was scanned by micro-computed tomography (μ-CT; Skyscan 1172, Skyscan, Aartselaar, Belgium) in cone-beam acquisition mode. The X-ray source was set at 50 kV and 200 μA with a 0.5-mm aluminum filter at 17.09 μm resolution. The exposure time was 1.2 s and 257 projections were acquired over an angular range of 180° (angular step; 0.7°). The image slices were reconstructed by using the Nrecon program (version 1.6.2.0, Skyscan, Aartselaar, Belgium) and bone volume was measured using the CT-Analyzer program (version 1.10.0.5, Skyscan, Aartselaar, Belgium). Three dimensional surface rendering images were made using the Mimics software version 14.0, imaging program (Materialise N.V, Leuven, Belgium).

### Histological analysis

All specimens were decalcified in a rapid decalcifying solution (Calci-Clear Rapid, National Diagnostics, GA, USA) for 10 days, and then embedded in paraffin and cut into 7-μm-thick serial slices. The sections were deparaffinized in xylene at room temperature for 20 min and then rehydrated through a graded series of alcohol solutions. The sections were then stained with hematoxylin and eosin.

### Statistics analysis

All experiments were repeated at least three times and statistical analysis was performed using one-way analysis of variance and Duncan’s multiple comparisons using the Graph Pad Prism 4 for Windows statistical software package (Graph Pad Software Inc., La Jolla, CA, USA). All the data presented is expressed as the mean ± SEM from three independent measurements. A *P*-value < 0.05 was considered to be statistically significant.

## Results

### BetA promotes osteogenic differentiation of MC3T3-E1 cells

**Fig. 1:**
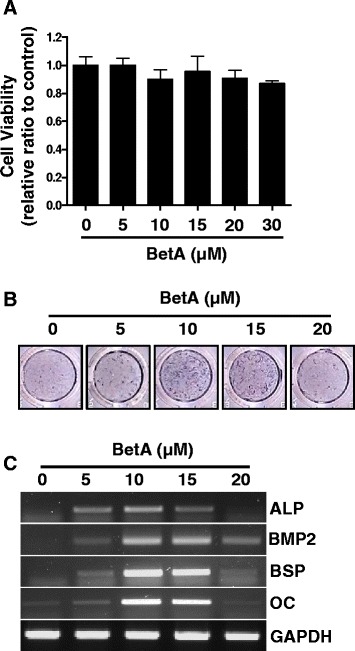
The effect of betulinic acid (BetA) on cell viability and osteogenic differentiation in MC3T3-E1 preosteoblast cells. **a** Cell viability assay. The MC3T3-E1 cells were cultured with different concentrations of BetA (0 μM to 30 μM) for 24 h, and cell viability was determined by WST-1. **b** ALP staining. The MC3T3-E1 cells were cultured in osteogenic medium containing different concentrations of BetA (0 μM to 20 μM) for 72 h, and then ALP staining was performed as in Materials and Methods. **c** RT-PCR analysis. The MC3T3-E1 cells were cultured as in (**b**). Total RNA was isolated and RT-PCR was performed with specific primers for ALP, BMP2, BSP and OC. The level of GAPDH was examined as a loading control. Representative data are shown. ALP, alkaline phosphatase. BSP, bone sialoprotein. OC, osteocalcin. *n* = 3

Prior to in vivo study, we first examined the effects of BetA on the cell viability and osteogenic differentiation in MC3T3-E1 cells. Results showed that BetA treatment at a concentration of up to 30 μM did not significantly reduce cell viability compared to the control group (Fig. 1a). Treatment with BetA, ranging from 5 to 15 μM, induced ALP activity and gene expression of the osteogenic markers, ALP, BMP2, BSP and OC (Fig. 1b and c). These results showed that although there was no effect on the cell viability at the concentrations used, BetA had the ability to promote osteogenesis.

### Treatment with a combination of BetA and BMP2 further enhances matrix mineralization

**Fig. 2:**
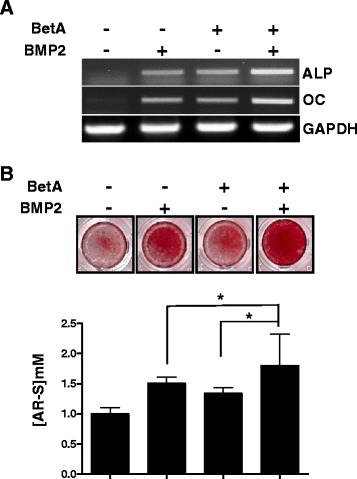
The effect of betulinic acid on BMP2-induced osteogenic differentiation and matrix mineralization in MC3T3-E1 preosteoblast cells. **a** RT-PCR analysis. The MC3T3-E1 cells were maintained with osteogenic medium containing BetA (10 μM) and/or BMP2 (200 ng/ml) for 10 days, and expression of osteogenic marker genes were measured by RT-PCR. **b** Mineralization assay. Cells were cultured as in (**a**), and were stained with alizarin red solution (upper panel). For quantification, the stains were eluted with 10 % cetylpyridinium and absorbance was measured by spectrophotometry (lower panel). ^*^, *p <* 0.05 compared to the indicated group. Representative data are shown. *n* = 3

BMP2 is well known for its osteogenic properties and is used clinically in the management of skeletal conditions requiring bone anabolism [25, 26]. Moreover, treatment with BetA potently increased the expression of BMP2 as well as that of other osteogenic markers (Fig. 1c). To further investigate the effects of BetA on BMP2-stimulated osteogenesis, MC3T3-E1 cells were cultured with BetA (10 μM) and/or BMP2 (200 ng/ml) for 10 days in osteogenic medium, and then gene expressions and matrix mineralization was analyzed. Results showed that treatment with a combination of BetA with BMP2 synergistically enhanced the gene expression of osteogenic markers, ALP and OC (Fig. 2a). Matrix mineralization was also significantly enhanced by a combination of BetA and BMP2 compared with groups treated with either BetA or BMP2 alone in a consistent fashion (Fig. 2b). These results suggest that BetA had an effect on osteogenic differentiation and greatly enhanced the BMP2-induced differentiation and mineralization in these cells.

### BetA enhances BMP2-dependent phosphorylation of Smad, ERK and p38

**Fig. 3:**
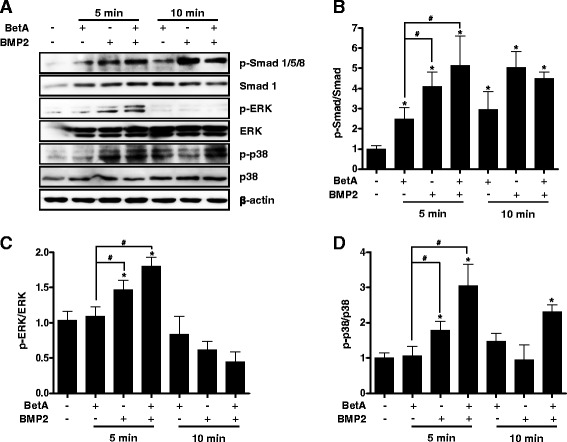
Effects of betulinic acid on phosphorylation of Smad, ERK and p38 in MC3T3-E1 cells. **a** Western blot analysis of phosphorylation of Smad, ERK and p38 before and after BetA (10 μM) and/or BMP2 (200 ng/ml) for 5 to 10 min. **b** to **d** Quantitative analysis. Phosphorylation of Smad1/5/8, ERK and p38 was quantified by using a LAS-4000 lumino-image analyzer system (Fujifilm, Japan). Each phosphorylation was normalized by the total amount of Smad1 (**b**), ERK (**c**) and p38 (**d**). ^*^, *p <* 0.05 compared to the control group. ^#^, *p <* 0.05 compared to the indicated group. Representative data are shown. *n* = 3

The BMP/Runx2 signal pathway is involved in BetA-mediated osteoblast differentiation [9]. Our results described above showed that BetA significantly increased BMP2-induced osteogenic differentiation (Fig. 2). In order to investigate the intracellular signaling pathways involved in this effect, we assessed whether BetA affected the levels of phosphorylation of Smad1/5/8 and MAPK (ERK and p38). Results showed that BetA stimulated the phosphorylation of Smad1/5/8 by about 1.5-2 fold compared to the control at 5 min and 10 min, respectively and increased the phosphorylation of p38 by about 0.4 fold compared with the control group at 10 min (Fig. 3a, b and d). Treatment with BMP2 treatment increased Smad1/5/8, ERK and p38 phosphorylation by about 3, 0.4 and 0.8 fold respectively at 5 min compared to the control. Treatment with a combination of BetA and BMP2 further enhanced the Smad1/5/8, ERK and p38 phosphorylation by about 4, 0.7 and 2 fold respectively at 5 min compared to the control (Fig. 3b-d). These results suggest that BetA could be a positive stimulator of the BMP signaling pathway and can enhance BMP2-induced osteogenic differentiation.

### BetA can enhance BMP2-induced ectopic bone formation

**Fig. 4:**
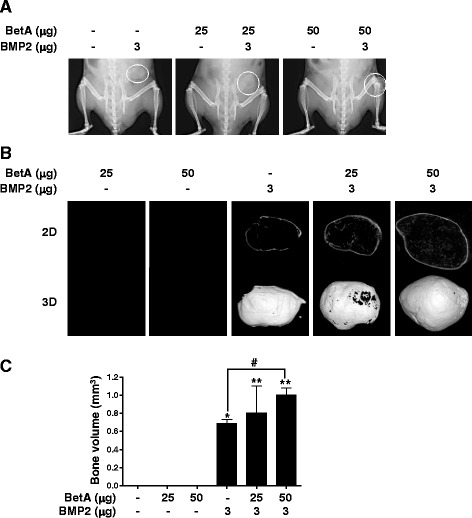
Radiographic study of the ectopic bone formation by BetA, BMP2 and BetA/BMP2 composite implants. BetA (25 or 50 μg) with or without BMP2 (3 μg) were administered with absorbable collagen sponges into the subcutaneous spaces in the back of mice. After 4 weeks, microradiographic analyses were performed. **a** Soft X-ray analysis. Ectopic bone formation was seen in BMP2- or BetA/BMP2-treated groups. Dotted circles in the right side of mice indicate the new ectopic bones. **b** Micro-computed tomography analysis. Each ectopic bone of (**a**) was isolated, and then scanned by μ-CT. The image slices were reconstructed three dimensionally as in Materials and Methods section. **c** Quantitative analysis. Ectopic bone volume was measured using a CT-Analyzer program. ^*^, *p <* 0.05, and ^**^, *p <* 0.01 compared to the control group (collagen sponge alone). ^#^, *p <* 0.05 compared to the indicated group. Representative data are shown. *n* = 5

**Fig. 5:**
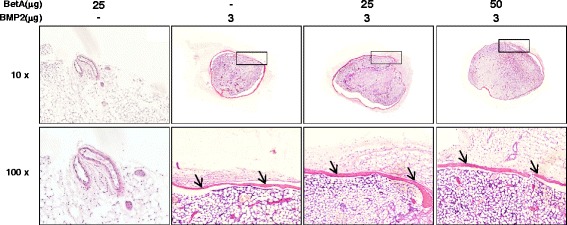
Histological examination of the ectopic bone with treatments of betulinic acid (25 or 50 μg), BMP2 (3 μg), and the combination of BetA and BMP2. All specimens used for radiographic analyses (Fig. 4) were formalin-fixed, paraffin-embedded and then cut into 7-μm-thick sections. The sections were then stained with hematoxylin and eosin. Low panels show the magnifying images of the box areas in upper panels. New bones are observed in BMP2- or BetA/BMP2-treated group as indicated with arrows. Microphotographs are shown at × 10 and × 100 magnification. *n* = 5

Based on the beneficial effect of treatment with a combination of BetA and BMP2 in vitro, we investigated whether such treatment could promote bone formation and improve callus bone parameters in vivo. In the present study, the effect of BetA on BMP2-induced ectopic bone formation was evaluated in following groups; control treatment (collagen sponge alone), BetA treatment (25, 50 μg), BMP2 treatment (1 μg, 3 μg), and combinational treatments of BetA/BMP2. BetA or/and BMP2 were delivered into the subcutaneous space on both sides of the backs of mice using an absorbable collagen sponge. At 4 weeks after implantation, new bone formation was analyzed by X-ray (2D) and high resolution μ-CT (3D) scanning. As shown in Additional file 1: Figure S1 and S4, the control and BetA treatment did not induce ectopic bone in the region of the implant. In contrast, implantation of BMP2 (1 or 3 μg) alone induced ectopic bone formation. In the group treated with the BetA (25, 50 μg) with BMP2 (1 μg), the amount of new bone tended to increase dependent on BetA doses (Additional file 1: Figure S1C). In addition, treatment with the combination of BetA (25, 50 μg) and BMP2 (3 μg) resulted in a significant increase in the volume of ectopic bone formation compared to treatment with BMP2 (3 μg) alone (Fig. 4c). Histological examination showed that the newly formed bone was an oval shape of single mass. Mineralized tissue was observed mostly at the periphery of the implants containing BMP2 alone or the combined composites. No significant adverse reaction observed in all groups (Fig. 5).

## Discussion

BMP2 is crucial for the development, induction, and differentiation of osteogenic cells as well as for the repair of bone defects [13, 23, 27]. However, there are disadvantages to the use of BMP2 for bone repair, including the high cost, rapid degradation, and edema [19, 20]. In order to overcome these limitations, the possibility of the use of a combination of BMP2 with biological or chemical agents offers a new surgical approach that might augment bone formation or even replace bone-grafting procedures [13]. Molecules including oxysterols [28], NELL1 [29], C-type natriuretic peptide [30], vascular endothelial growth factor (VEGF) [31], and parathyroid hormone-related peptide (PTHrP) [32] have been shown to be either synergistic with or supportive of BMP induction of osteogenic differentiation in vitro. Furthermore, in vivo studies have also shown synergistic enhancement in bone formation when BMPs are combined with VEGF and NELL1 [29, 31]. BetA was also reported to be a potent osteogenic molecule acting through the BMP/Runx2 and β-catenin signaling pathways [9]. The present study was conducted to prove the hypothesis that treatment with a combination of BetA and BMP2 will be more effective for osteogenic differentiation and bone formation than BMP2 treatment alone. Our results showed that BetA increased the gene expression of osteogenic markers as well as ALP activity (Fig. 1). Treatment with a combination of BetA and BMP2 significantly increased osteogenic marker gene expression and matrix mineralization compared to treatment with BMP2 alone, indicating a synergistic effect on osteogenic differentiation (Fig. 2) and suggesting that BetA may influence BMP2 mediated osteoblast functions.

In order to further understand the molecular mechanism involved in BetA potentiation of BMP2 action, since there are no known receptors for BetA, we investigated the possibility that BetA may have an impact on signaling pathways that can crosstalk with BMP signaling. The phosphorylation of Smad1/5/8 and MAPK signaling pathways, which have been shown to directly stimulate bone formation and osteoblast differentiation, are usually triggered by BMPs [22, 24]. In the present study, BetA treatment did indeed increase the phosphorylation of Smad1/5/8, ERK and p38. Furthermore, combination of BetA and BMP2 enhanced the Smad1/5/8 and p38 phosphorylation even more as compared with each one alone (Fig. 3). These results suggest that BetA stimulates the BMP2 action on osteogenic differentiation through the Smad1/5/8 and p38 signaling pathways. However, we did not identify yet whether BetA can affect BMP2-induced bone formation via immobilizing the BMP2 in the absorbable collagen sponge, or via inhibiting the degradation of BMP2, or via increasing binding of BMP2 to its receptors, or via activating other signal pathways. Further studies are still needed for determining the exact mechanism.

In this study, we have demonstrated for the first time the effect of treatment with BetA and with a combination of BetA and BMP2 in an animal model. The subcutaneous ectopic bone formation increased after treatment with either BMP2 alone or with the combination of 25 μg BetA and 3 μg BMP2 in mice (Fig. 4). The combination of BetA and BMP2 resulted in a bone volume approximately 30 % higher than that observed when BMP2 alone was used (Fig. 4c). Increasing the concentration of BetA up to 50 μg under maintaining BMP2 at 3 μg enhanced the volume of newly formed bone by approximately 70 % compared to that observed using 3 μg BMP alone (Fig. 4c). The data indicate that BetA and BMP2 acts synergistically to induce more bone formation than does BMP2 alone. However, when 25 or 50 μg of BetA was treated with 1 μg of BMP2, ectopic bone formation merely tended to increase compared to the BMP2 alone group; the differences between groups did not reach statistical significance (Additional file 1: Figure S1). These findings suggest that doses or ratio of BetA and BMP2 combination would be also crucial for synergistic bone formation in vivo.

The present study provides beneficial evidence that combination of BetA and BMP2 can enhance osteogenesis. Nevertheless, there are some limitations in this study that should be noted. First, combinatory effects of BetA and BMP2 on bone formation were examined just in an ectopic bone formation model. Because BetA and BMP2 will be mostly applied to bony defects for the purpose of therapy, the combinatory effects have to be approved more in orthotopic bone formation models. Second, our study did not determine the best dosage for combination, which might be significant to develop a therapeutic strategy without dose-related side effects. In addition, this study did not include the optimal timing of application (sequential or simultaneous administration) and proper carriers. For therapeutic uses of the combination, further studies are needed with more specific experimental conditions and various animal models.

Taken together, our results showed that BetA up-regulates the expression of BMP2-induced osteogenic marker genes and mineralized matrix deposition in preosteoblastic cells, which results in augmentation of bone formation through activating the Smad 1/5/8 and p38 signaling pathways. BetA could be described as a positive stimulator for BMP2-induced ectopic bone formation.

## Conclusions

In order to improve the bone formation by BMPs, several scientists have investigated the use of BMPs in combination with other agents. The present study, using the ectopic bone formation model, indicates that BetA acts synergistically with BMP2 to accelerate bone formation. Thus, combination therapy with BetA and BMP2 may be of great benefit in fracture healing and for patients undergoing reconstructive surgery.

## Additional files

Additional file 1: Figure S1. Radiographic study of the ectopic bone formation after treatment of BetA, low dose of BMP2 and BetA/BMP2. BetA (25 and 50 μg) with or without BMP2 (1 μg) was administered with absorbable collagen sponges into the subcutaneous spaces in the back of mice, as in Fig. 4. After 4 weeks, ectopic bone formation was analyzed by Soft X-ray (A), μ-CT (B), and quantified by using a CT-Analyzer program (C). Dotted circles in (A) indicate the new ectopic bones. ^******^, *p <* 0.01 compared to the control group (collagen sponge alone). NS, not significant. Representative data are shown. *n* = 5. (PDF 114 kb)
