# Supplementary figures and images for: Betulinic acid synergically enhances BMP2-induced bone formation via stimulating Smad 1/5/8 and p38 pathways
Source: J Biomed Sci. 2016 May 17;23:45. doi: 10.1186/s12929-016-0260-5 (PMC4869197; doi:10.1186/s12929-016-0260-5)

# Supplementary Fig. 1

**A**

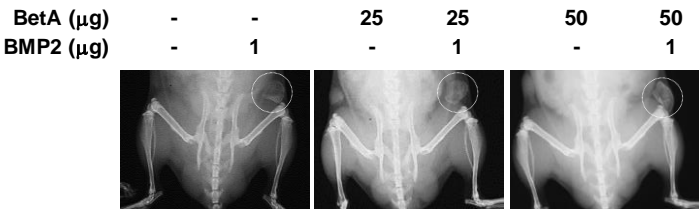

**B**

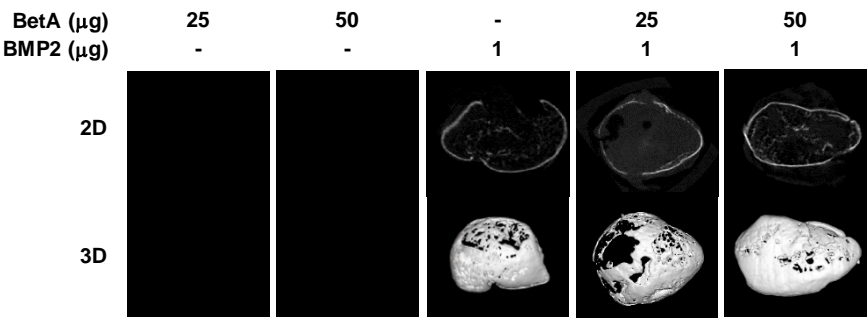

**C**

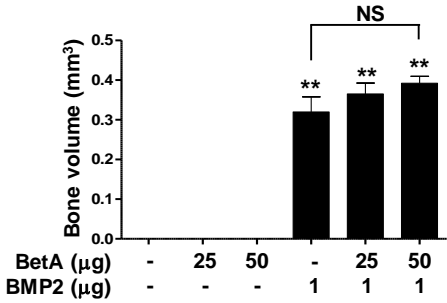

Supplement: Additional file 1: Figure S1. — Radiographic study of the ectopic bone formation after treatment of BetA, low dose of BMP2 and BetA/BMP2. BetA (25 and 50 μg) with or without BMP2 (1 μg) was administered with absorbable collagen sponges into the subcutaneous spaces in the back of mice, as in Fig. 4. After 4 weeks, ectopic bone formation was analyzed by Soft X-ray (A), μ-CT (B), and quantified by using a CT-Analyzer program (C). Dotted circles in (A) indicate the new ectopic bones. **, p < 0.01 compared to the control group (collagen sponge alone). NS, not significant. Representative data are shown. n = 5. (PDF 114 kb) [file 12929_2016_260_MOESM1_ESM.pdf]
